# Supplementary material for: MetaQTL: a package of new computational methods for the meta-analysis of QTL mapping experiments
Source: BMC Bioinformatics. 2007 Feb 8;8:49. doi: 10.1186/1471-2105-8-49 (PMC1808479; doi:10.1186/1471-2105-8-49)
Supplement: Additional File 7 — MetaQTL Package : jar file and tutorial. This Zip archive contains both the MetaQTL JAR file and the files of the tutorial. [file 1471-2105-8-49-S7.zip › org.inra.metaqtl/doc/org/metaqtl/MetaQtlResult.html]

MetaQtlResult


|  |  |  |
| --- | --- | --- |
| **Overview** \| **Package** \| **Class** \| **Use** \| **Tree** \| **Deprecated** \| **Index** \| **Help** | |  |
| **PREV CLASS**   **NEXT CLASS** | **FRAMES**    **NO FRAMES**     **All Classes** |  |
| SUMMARY: NESTED \| FIELD \| CONSTR \| METHOD | DETAIL: FIELD \| CONSTR \| METHOD |  |


---


## org.metaqtl Class MetaQtlResult

```
java.lang.Object
  org.metaqtl.MetaQtlResult
```

---

``` public class MetaQtlResult extends java.lang.Object ```

A `MetaQtlResult` is used to store the
results of a QTL meta analysis which has been performed
for a given trait group on a given chromosome.

---

| **Field Summary** | |
| --- | --- |
| `EMResult[]` | `clusterings`             The meta qtl clusterings. |
| `int` | `nmqc`             The number of meta qtl clusterings. |
| `int` | `nqtl`             The number of QTL |
| `int[]` | `qtlIdx`             The indices of QTL which are implied in the analysis. |
| `double[]` | `sd`             The QTL position standard deviations. |
| `java.lang.String` | `trait`             The name of the trait. |
| `Tree` | `tree`             The qtl tree |
| `double[]` | `x`             The QTL positions. |


**Constructor Summary**

`MetaQtlResult()`

`MetaQtlResult(java.lang.String trait,
int qtlNumber)`


| **Method Summary** | |
| --- | --- |
| `EMResult` | `getBestClustering(int criterion)`             This methods returns the best models for each criterion defined to select the optimal number of clusters per trait. |
| `EMResult` | `getClustering(int k)`             Returns the Qtl clustering result with `k` clusters. |
| `double[]` | `getCritDelta(java.lang.String criterion, int[] K)` |
| `java.util.Iterator` | `getCriteria()` |
| `double[]` | `getCritValues(java.lang.String criterion, int[] K)` |
| `double[]` | `getCritWeights(java.lang.String criterion, int[] K)` |
| `int[]` | `getKValues()` |
| `Qtl[]` | `getMetaQtl(int k)` |
| `Tree` | `getQtlTree()` |
| `java.lang.String` | `getTraitName()`             Returns the name of the trait cluster for this result. |
| `double[]` | `getUSD(EMResult best, int kmin, int kmax, java.lang.String criterion)` |
| `void` | `setClusterings(EMResult[] results)` |
| `void` | `setClusterings(java.util.List list)` |
| `void` | `setQtlIdx(int[] qtlIdx)`             Set the indices of the Qtl which are implied in this result. |
| `void` | `setSD(java.lang.Double[] sd)`             Set the standard deviations of the Qtl. |
| `void` | `setTree(Tree tree)` |
| `void` | `setX(java.lang.Double[] xx)`             Set the observed positions of the Qtl. |

**Methods inherited from class java.lang.Object**

`clone, equals, finalize, getClass, hashCode, notify, notifyAll, toString, wait, wait, wait`

**Field Detail**

### trait

```
public java.lang.String trait
```

:   The name of the trait.

---


### nqtl

```
public int nqtl
```

:   The number of QTL

---


### nmqc

```
public int nmqc
```

:   The number of meta qtl clusterings.

---


### qtlIdx

```
public int[] qtlIdx
```

:   The indices of QTL which are implied
    in the analysis.

---


### x

```
public double[] x
```

:   The QTL positions.

---


### sd

```
public double[] sd
```

:   The QTL position standard deviations.

---


### clusterings

```
public EMResult[] clusterings
```

:   The meta qtl clusterings.

---


### tree

```
public Tree tree
```

:   The qtl tree


**Constructor Detail**

### MetaQtlResult

```
public MetaQtlResult()
```

---


### MetaQtlResult

```
public MetaQtlResult(java.lang.String trait,
                     int qtlNumber)
```

**Parameters:**: `qtlNumber` -


**Method Detail**

### getClustering

```
public EMResult getClustering(int k)
```

:   Returns the Qtl clustering result with `k`
    clusters.

    :   **Parameters:**: `k` - **Returns:**

---


### setClusterings

```
public void setClusterings(java.util.List list)
```

:   **Parameters:**: `emResultList` -

---


### setClusterings

```
public void setClusterings(EMResult[] results)
```

:   **Parameters:**: `emResults` -

---


### setQtlIdx

```
public void setQtlIdx(int[] qtlIdx)
```

:   Set the indices of the Qtl which are implied in
    this result.

    :   **Parameters:**: `qtlIdx` - the indices of the Qtl.

---


### setX

```
public void setX(java.lang.Double[] xx)
```

:   Set the observed positions of the Qtl.

    :   **Parameters:**: `xx` -

---


### setSD

```
public void setSD(java.lang.Double[] sd)
```

:   Set the standard deviations of the Qtl.

    :   **Parameters:**: `xx` -

---


### setTree

```
public void setTree(Tree tree)
```

:   **Parameters:**: `tree` -

---


### getBestClustering

```
public EMResult getBestClustering(int criterion)
```

:   This methods returns the best models for each criterion
    defined to select the optimal number of clusters per
    trait.

    :   **Returns:**

---


### getTraitName

```
public java.lang.String getTraitName()
```

:   Returns the name of the trait cluster for this result.

    :   **Returns:**: the name of the trait cluster.

---


### getKValues

```
public int[] getKValues()
```

:   **Returns:**

---


### getCriteria

```
public java.util.Iterator getCriteria()
```

:   **Returns:**

---


### getCritValues

```
public double[] getCritValues(java.lang.String criterion,
                              int[] K)
```

:   **Parameters:**: `criterion` - **Returns:**

---


### getCritDelta

```
public double[] getCritDelta(java.lang.String criterion,
                             int[] K)
```

:   **Parameters:**: `criterion` - **Returns:**

---


### getCritWeights

```
public double[] getCritWeights(java.lang.String criterion,
                               int[] K)
```

:   **Parameters:**: `criterion` - **Returns:**

---


### getUSD

```
public double[] getUSD(EMResult best,
                       int kmin,
                       int kmax,
                       java.lang.String criterion)
```

:   **Parameters:**: `best` - **Returns:**

---


### getQtlTree

```
public Tree getQtlTree()
```

:   **Returns:**

---


### getMetaQtl

```
public Qtl[] getMetaQtl(int k)
```

:   **Returns:**


---


|  |  |  |
| --- | --- | --- |
| **Overview** \| **Package** \| **Class** \| **Use** \| **Tree** \| **Deprecated** \| **Index** \| **Help** | |  |
| **PREV CLASS**   **NEXT CLASS** | **FRAMES**    **NO FRAMES**     **All Classes** |  |
| SUMMARY: NESTED \| FIELD \| CONSTR \| METHOD | DETAIL: FIELD \| CONSTR \| METHOD |  |


---
